# Ethical challenges in reproductive donation tourism: a qualitative study of clinicians’ perspectives in Iran

**DOI:** 10.1186/s12910-026-01547-8

**Published:** 2026-07-01

**Authors:** Nasrin Sarafraz, Faezeh Ghafoori

**Affiliations:** 1 Faculty of Nursing and Midwifery, Islamic Azad University, Larestan, Iran; 2 Nursing and Midwifery Department, Social Determinants of Health Research Center, Saveh University of Medical Sciences, Saveh, Iran

**Keywords:** Fertility, Medical tourism, Oocyte donation, Qualitative study, Ethics

## Abstract

**Background:**

Reproductive donation tourism is a complex phenomenon with profound ethical implications which also affects Iran. However, studies in this context are limited. This study explores the ethical challenges associated with donor-based reproductive tourism from the perspective of Iranian clinicians, focusing on how they interpret these challenges within Iran’s legal, religious, and institutional context.

**Methods:**

A conventional content analysis study was used to explore the ethical concerns in donation-related reproductive tourism perceived by clinicians in Iran. Twenty-two experts who were actively involved in the field of infertility treatment, were recruited purposefully from ART centers in Tehran and the Ministry of Health of Iran in 2025. Data collection was done through in-depth, semi-structured interviews. Analysis was carried out by MAXQDA.24 software and the Graneheim and Lundman approach. A conceptual model was also proposed to explain the interaction of challenges involved in this phenomenon.

**Results:**

Data analysis resulted in four themes: (1) Legal and structural heterogeneity of countries; (2) Clinical uncertainty and physical and genetic concerns in reproductive donation; (3) Synergistic moral, psychological, identity, social, and cultural-religious implications and secrecy in reproductive donation; (4) Justice, commodification, exploitation and demand in fertility tourism. These challenges can raise concerns related to justice, exploitation, commodification, and the rights of donor-conceived children.

**Conclusion:**

This treatment method in Iran is surrounded by multidimensional and interwoven concerns that mutually reinforce each other. These findings indicate that reproductive donation tourism is not merely a legal and clinical issue but one that raises significant ethical concerns, requiring careful consideration of fundamental principles of medical ethics in both national and international policy-making. Therefore, developing and sharing integrated international legal and ethical frameworks to support ethically responsible and regulated reproductive care, is essential.

**Supplementary Information:**

The online version contains supplementary material available at https://doi.org/10.1186/s12910-026-01547-8.

## Introduction

Infertility is a significant life challenge that, under social and familial pressures, can seriously compromise couples’ well-being and life satisfaction [1]. Thus, more people have started resorting to assisted reproductive technologies and third-party donation [2].

Third-party donation in infertility treatment-one of the most significant achievements of advanced reproductive technologies-includes the use of gametes (sperm or oocytes), portions of gametes (such as oocyte cytoplasm), reproductive tissues, embryos, or uterine surrogacy [3]. Although these techniques broaden the possibilities of childbearing for infertile couples, cross-border reproductive donation may raise additional ethical and regulatory complexities due to differences in legal frameworks, religious regulations, anonymity policies, socioeconomic conditions, and varying conceptions of kinship and parenthood across countries [4–6]. This phenomenon is known as “reproductive tourism”, which is defined as the movement of individuals across borders to access infertility treatments unavailable or restricted in the home country [7, 8].

Evidence indicates that approximately 20,000 to 25,000 couples seek infertility treatment abroad every year [8]. Iran is one of the few countries that has become a major destination for infertile couples from neighboring and regional countries. The prohibition of donation in many countries seems to have increased the cross-border movements of infertile couples to countries like Iran [6, 9]. Additionally, the relatively low cost combined with high-quality medical services has been a contributing factor that has increased the demand for such treatments among Arabic countries [6].

Despite the rapid growth of this phenomenon, a review of the existing literature shows that most studies about donation are either narrative or conceptual and focus on religious issues [10–12]. Only a limited number of studies have addressed reproductive tourism, and mostly studies have basically focused on medical tourism issues [8, 13], the main attention of researchers has so far been drawn to fertility tourism for the use of ART [9, 14], and fertility tourism related to donation within the Iranian context has been given less attention. For example, HashemiNasab and Garousi (2024) described surrogacy in Iran as a complex social network shaped by familial relationships, gender expectations, and social interactions [15]. Similarly, Ghane-Mokhallesouni and colleagues (2021), through interviews with surrogate mothers in Yazd, identified challenges including emotional burden, and social judgment [16]. Therefore, much of the available knowledge in this area comes from studies conducted in other countries [5, 17], which, although informative, may not fully reflect the sociocultural, religious, and regulatory context of Iran. This highlights the need for context-specific research focusing on Iranian clinicians’ perspectives.

Given this research gap, a qualitative approach is warranted to explore the experiences and perspectives of clinicians involved in fertility care in Iran regarding donation-based reproductive tourism. This approach allows for an in-depth understanding of the multidimensional and ethical challenges associated with this phenomenon in the Iranian context. Therefore, this study addresses the following research question: What ethical challenges do clinicians perceive in cross-border donor-assisted reproductive tourism, and how do they interpret these challenges within Iran’s legal, religious, and institutional context?

While previous studies have often focused on religious aspects of third-party donation, its ethical challenges—particularly in relation to principles such as autonomy, justice, non-maleficence, and the rights of donor-conceived children—remain underexplored in the Iranian context. This lack of emphasis on clinicians’ ethical perspectives represents a significant gap in the existing literature.

## Methods

### Study Design

This qualitative study was conducted according to a conventional content analysis approach. This approach, enables the researcher to interpret findings through a systematic presentation of data in the form of words and themes, entailing comprehension, interpretation, and conceptualization of the underlying meanings within the qualitative data [18].

In this study, 22 clinicians (including 15 women and 7 men) were recruited between January and October 2025 using purposive maximum variation sampling. Participants were selected to ensure diversity in age, gender, professional discipline, institutional affiliation, and years of experience in infertility care (see Table 1).

**Table 1:** Characteristics of the study participants

Characteristics	*n*	%
Age range (years)		
25–30 30–40 40–50 50–55	1 8 10 3	4.5 36.4 45.5 13.6
Gender		
Female Male	15 7	68.2 31.8
Education		
Medical Ethics General Medicine Reproductive Biology (PhD) Reproductive Health (PhD) Gynecology (Infertility) Embryology Urology Midwifery Nursing	3 2 3 3 4 2 2 2 1	13.6 9.1 13.6 13.6 18.2 9.1 9.1 9.1 4.5
Job (Professional role)		
ART Clinicians Policymakers	16 6	72.7 27.3
Work experience (years)		
1–5 5–10 10–15 15–20 20–25	4 8 5 4 1	18.2 36.4 22.7 18.2 4.5

*PhD* Philosophy of Degree

All participants were working in ART centers in Tehran, Iran (including Imam, Shariati, Arash, Yas hospitals) and 6 of them were working in Ministry of Health, too. To facilitate participant recruitment, official correspondence was first conducted with the relevant universities affiliated with the ART centers, during which the study aims, the researcher’s affiliation, and the ethical approval code were formally introduced. Following this process, permission letters were obtained from the deputy of treatment of the affiliated universities to access the infertility treatment centers.

The researcher (FG) then visited the ART centers, where eligible clinicians meeting the inclusion criteria (at least one year of professional experience in infertility treatment or reproductive donation services and willingness to participate in the study) were identified in collaboration with the centers’ administration. The participants were contacted directly by the researcher, informed about the aims and procedures of the study, and invited to participate voluntarily. Interviews were subsequently scheduled at times and locations convenient for participants who agreed to participate.

### Data collection

Data were collected through individual, semi-structured, face-to-face interviews. Sampling continued until data saturation was reached— saturation was determined after 20 interviews, when no new codes emerged in two consecutive interviews and was confirmed through team discussion.

Each interview lasted approximately 30 to 45 min, depending on participant conditions, and each participant was interviewed once. In total, 22 interviews were conducted at convenient times and locations for the participants. Six interviews took place at the Ministry of Health, while the remaining interviews were conducted privately in ART centers, typically in participants’ workplaces. Interviews began with broad and general questions, followed by more detailed probes.

One researcher, with clinical background in infertility care, performed all interviews. The interviewer’s clinical background in infertility care likely facilitated rapport and promoted openness among participants due to shared professional experience. At the same time, this positionality may have influenced interview interactions and data interpretation. Reflexive journaling and team discussions were used to mitigate potential bias. The interviewer engaged in reflexive practices throughout the study and remained aware of potential preconceptions and disciplinary perspectives. The interviewer attempted to bracket prior assumptions during data collection and maintained a neutral and non-judgmental stance, allowing participants to freely express their views. Some participants held dual institutional roles in both ART centers and the Ministry of Health and Medical Education. This dual positioning may have influenced their perspectives by combining clinical experiences with policy and regulatory considerations, which was considered during data interpretation and team discussions. A semi-structured interview guide was also used to ensure consistency across interviews and to reduce interviewer bias. The semi-structured interview guide was developed by the research team based on a review of the relevant literature and expert consultation. The English version of the interview guide is provided as Supplementary File 1.

The interview guide explored participants’ perceptions of legal, religious, and institutional considerations in reproductive donation, without attempting to systematically analyze formal legal texts or religious rulings.

### Data analysis

Data analysis was performed according to the approach described by Graneheim and Lundman [19]. The analysis started immediately after the first interview. All interviews were audio-recorded. The audio file was transcribed verbatim into Microsoft Word after each interview. MAXQDA 24 software was utilized to manage the coding process. Initial codes were extracted after repeated listening to the recordings. Then, by looking for similarities in the codes, subthemes and themes were established. Finally, four main themes emerged.

All transcripts were independently reviewed and coded by two researchers. Disagreements in coding were resolved through discussion and consensus. In cases where consensus was not reached, a third senior researcher, who was not involved in the initial coding process, was consulted to adjudicate discrepancies.

To ensure the credibility of the findings, the researchers maintained prolonged and continuous engagement with the data through transcription, repeated reading of transcripts, coding, and iterative analysis. Member checking and peer checking were also employed to enhance the trustworthiness of the findings. Member checking consisted of returning coded interview transcripts to five participants to assure agreements among researchers and participants.

Peer checking included having two experts’ review and comment on the coding and categorizing. Two experienced researchers independently reviewed the extracted codes and themes to establish confirmability. Additional follow-up questions were asked to participants to further develop the accuracy of the interpretations. Transferability was enhanced through purposive sampling with maximum demographic variation, as well as through the detailed descriptions provided regarding the data collection and data analysis procedures.

All interviews were conducted under similar conditions and by the same researcher to maintain consistency.

### Ethical consideration

This study was approved from the Ethics Committee of Saveh University (Code: Code IR.SAVEHUMS.REC.1403.046). The principal author was affiliated with Saveh University at the time of the study. However, Tehran was purposefully selected as the study setting because ART centers authorized to provide oocyte and embryo donation, are in this city. In addition, policymakers and experts in the field of infertility are based at the Ministry of Health and Medical Education in Tehran.

Such specialized infertility centers and policy-level stakeholders are not available in Saveh; therefore, interviews were carried out in Tehran. The permissions and necessary coordination were obtained from the Tehran University of Medical Sciences and, subsequently, from its ART centers, including Imam Khomeini, Shariati, and Arash hospitals.

All participants were fully informed about the aims and procedures of the study. Written informed consent was obtained from all participants prior to the interviews. Participants were assured of confidentiality, anonymity, and their right to withdraw at any time without consequences.

## Results

The participants’ characteristics are shown in Table 1. Four themes emerged: legal and structural heterogeneity of countries; clinical uncertainty and physical and genetic concerns in reproductive donation; synergistic moral, psychological, identity, social, and cultural-religious implications and secrecy in reproductive donation; justice, commodification, exploitation and demand in fertility tourism (see Table 2).

**Table 2:** Themes, sub-themes and example of codes

Themes	Examples of codes	Frequency (*n*, %)
1. Legal and structural heterogeneity of countries		
1.1. Legal contradictions between the origin and destination countries	legal gaps regarding single persons; age-related service restrictions differ between the two countries	19 (86.4%)
1.2. Legal ambiguities in the origin and destination countries	ambiguity of descent and succession, uncertainty over the child’s citizenship status in the country of destination; children rights in the destination country after the death of social parents; child’s right to know their genetic parents	9 (41%)
1.3. Differences in medical standards between the origin and destination countries	differences in criteria for high-quality embryos for transfer; differences in permissible donation criteria in the two countries; differences in services and technologies in the two countries	4 (18.2%)
1.4. Lack of financial transparency	uncertainty regarding compensation for gamete and surrogate donation, differences in methods of payment for donation; differences in methods of payment for pregnancy-related treatments	8 (36.4%)
1.5. Weaknesses in data registration and exchange systems	inaccurate data registration, lack of a shared database system between countries; weak in reporting systems	18 (81.8%)
2. Clinical uncertainty and physical and genetic concerns in reproductive donation		
2.1. Scientific uncertainty	disagreement on age limits for using donations; disagreement on the number of gametes donated by a woman or man; disagreement on donor selection and screening criteria; disagreement over frozen gametes and embryos storage period	15 (68.2%)
2.2. Donor physical harm	risks of long-term hormonal treatment in female donors; invasive nature of ovarian hyperstimulation; risks of anesthesia; ovarian puncture for follicle retrieval	15 (68.2%)
2.3. Perceived genetic and demographic concerns related to reproductive donation	concerns regarding genetic diversity and donor traceability; risk of emerging new genetic diseases; male embryos selection; destruction of female embryos; disruption of the country’s sex ratio	12 (54.5%)
3. Synergistic moral, psychological, identity, social, and cultural-religious implications and secrecy in reproductive donation		
3.1. Parent-child identity harms	damaged identity in children; identity conflict in donor-conceived children; genetic mother versus social mother; parents’ concern about disclosure of identity	20 (91%)
3.2. Concerns about the child’s future	concerns on child abuse in the destination country; concerns about the future of children in the destination country; child’s adaptation to the destination society; concerns about the child’s social status in future	7 (31.8%)
3.3. Psychological distress in parents and child	feelings of guilt; emotional attachment of the surrogate mother to the child in utero; child’s emotional attachment to social parents; disruption in the concept of motherhood	15 (68.2%)
3.4. Damaged family relations	non-acceptance of child from another country by the parents’ family; rejection of the child, and sometimes the recipients, by family of parents	17 (77.3%)
3.5. Multiple social stigmas	infertility stigma; negative perception of donor women among family and society; social judgment or rejection of the donor-conceived child; stigma of tourism and leisure	19 (86.4%)
3.6. religious ambiguities and disagreements	ambiguity in child’s religious identity; child’s mahram status with recipients ambiguous; child’s religious ambiguous with other recipient family members; religious differences between origin and destination countries	10 (45.5%)
3.7. Parental and child concealment	secrecy of the method of child birth; secrecy of donation; secrecy of the genetic parents from the child	13 (59.1%)
4. Justice, commodification, exploitation and demand in fertility tourism		
4.1. Inequality in access wealthy vs. low-income countries	high cost of treatment; limited insurance coverage for services; luxury nature of donation services; commercialization of low-income women’s bodies; limited access to services due to use by foreign individuals; inability to afford treatment in home country as a driver for migration	19 (86.4%)
4.2. Export-import markets for donation	Intermediation; communication through agencies; black market; market for buying and selling gametes and embryos; sale of gametes by celebrities	9 (41%)
4.3 Positive perceptions and growing demand for fertility tourism	Positive view of fertility tourism; Acceptance of fertility tourism; Increasing demand for fertility tourism	20 (91%)

Percentages are based on 22 participants. Multiple challenges and concerns were stated; therefore, totals may exceed 100%

It should be mentioned that all findings reflect participants’ perceptions, reported experiences, and interpretations rather than verified empirical facts. In addition, the frequencies reported in the Results section are intended to provide a descriptive indication of the distribution of participants’ views and should not be interpreted as generalizable findings.

### Theme 1. Legal and structural heterogeneity of countries

Nineteen participants, based on their perception of Iranian legal and religious (fiqh) frameworks and institutional regulations in ART centers, underlined that the regulatory inconsistencies between the country of origin and destination are the most important factor shaping—and at the same time complicating—donation tourism. According to them, a difference in law related to the eligible recipient, such as single persons or age restrictions, compels couples to travel for the purpose of evading such limitations. Seven participants expressed concern that legal inconsistencies between source and destination countries may contribute to political or diplomatic tensions in some circumstances. For instance, one participant noted that: “*Germany has restrictions on gamete donation*,* which may encourage some couples to travel to other countries seeking these services”.*

Nine participants noted that legal inconsistencies may create difficulties in obtaining citizenship. Ambiguity, with surrogacy, was highlighted; as one participant reported that: “*I’ve witnessed a case of a child who had not gained citizenship from either the source or the destination country*,* and the family faced difficulties leaving Iran*”.

Four participants also perceived that some legal difficulties arise from varying definitions and medical parameters in different countries. For example, one participant stated on the issue of age limits for donation: *“some countries*,* like Iran*,* put the age limit for egg donation at 30 years*,* but some other countries set a different age limit”.*

Eleven participants described such inconsistencies in standards as possibly leading to patient misunderstandings, legal objections, and complaints in the source country. Eight participants expressed concerns that differences in compensation and lack of standardized oversight across countries may create conditions that lead to economic irregularities.

Thirteen of the interviews revealed that the existing managerial structures in the Ministry of Health and infertility centers are deficient in required transparency and coordination. Four participants stated that each center has its own rules, and there is no national standard for collecting and transferring data.

Eighteen participants highlighted that lack of coordination between countries limits the reliability and applicability of collected data. Without agreements and defined standards with the source or destination country, the data can never be considered reliable.

It should be mentioned that participants’ responses reflected their perceptions of multiple overlapping normative frameworks, including formal state regulations, religious interpretations (fiqh), and institutional practices within ART centers.

### Theme 2. Clinical uncertainty and physical and genetic concerns in reproductive donation

Fifteen participants attributed the confusion among physicians and even patients at the time of decision-making to a lack of clear scientific protocols in Iran. For instance, one participant said that: “*we don’t have specific guidelines showing how many times we can retrieve eggs from one woman… This complicates the process*,* and each infertility center applies its own discretionary approach*”.

Fifteen participants reported that treatment centers occasionally disagree on the quality of gametes and embryos. They said that such disagreements make clinical decisions more difficult, create anxiety for patients, and may reduce confidence in the treatment process.

A substantial subtheme that was merged here was physical harm to donors. Five participants stated that egg donors are vulnerable to two kinds of physical dangers: pharmacological and surgical. Ten participants mentioned that certain donors suffer physical alterations induced by ovulation-stimulating drugs or other hormonal treatments, which cause mood swings, weight gain, and chronic fatigue. They stated that some donors complain of various complications regarding the surgical donation process, such as pain, bleeding, and infection.

Twelve participants expressed concerns regarding potential long-term genetic implications at the population level. Eight participants raised concerns regarding possible future genetic risks associated with repeated use of a limited number of donors. These views reflected participants’ anticipatory concerns rather than documented population-level evidence. They suggested that some form of regulation regarding repeated donation and cross-border donation practices might be considered as a preventive measure.

Three participants also expressed concern that preferential embryo selection is viewed as a factor that might influence sex-ratio patterns over time, although these concerns were speculative and not based on observed demographic evidence. Most participants stated that these challenges influenced clinicians’ decision-making in practice by requiring reliance on individual judgment rather than standardized protocols.

### Theme 3. Synergistic moral, psychological, identity, social, and cultural-religious implications and secrecy in reproductive donation

Twenty participants described international fertility tourism for donation services as involving interrelated moral, psychological, identity-related, social, and cultural-religious concerns rather than isolated dimensions. They described these domains as mutually reinforcing, with psychological, social, identity-related, and religious concerns interacting to intensify uncertainty and collectively leading to secrecy.

A manager at the Ministry of Health reported receiving several complaints or inquiries at their office from donor-conceived children who seek their biological parents. He explained that: “*others only discover the truth when their parents die*,* thus causing confusion*,* and others still through family disputes*”. This psychological burden was perceived as contributing to concerns about maintaining secrecy within family and social contexts.

Eighteen professionals reported ongoing stress and tension among recipient parents. Participants reported that many recipient parents were worried about disclosure of biological parentage, a fact even infertility specialists acknowledged. In contrast, ten participants were concerned about child’s right to identity (i.e., the right to access information about biological origins and genetic parentage), and one of them stated that: “*child have a right to know biological parents*”.

Seventeen participants reported concerns regarding family acceptance of donor-conceived children, especially when services were obtained abroad, were another concern for couples receiving donations. As one specialist shared that: “*I have been working in an infertility center for 16 years. I have seen women who sought children to preserve their marriage*,* but after having a child through surrogacy*,* even their own families abandoned them…*”. He told that: “*these identity and psychological challenges were perceived to potentially affect parent–child and spousal relationships*,* contributing to familial tension.*”

Five participants reported awareness of cases in which donor-conceived individuals later experienced confusion or distress upon learning about their conception, which participants believed influenced parents’ decisions toward secrecy.

Nineteen participants also emphasized social stigmas. They claimed that the term fertility tourism itself holds stigma, with its connotations of leisure/pleasure when couples are forced to travel due to legal or other compelling reasons. Thirteen participants reported that these multiple stigmas of infertility, donation, and tourism, frequently resulted in concealing the facts.

Ten participants explained that child’s mahram status (i.e., the religious/legal non-marriageable kinship relationship in Islamic jurisprudence) with recipients is ambiguous, and one of them emphasized: “*this matter is important for most parents*”. This ambiguity was perceived to increase hesitation among couples regarding disclosure, especially in cross-border arrangements where legal and religious frameworks may conflict.

Seven participants suggested that cross-border reproductive arrangements may raise concerns regarding the child’s social status and integration in the destination country. One participant reported: “*observing cases where donor-conceived children were allegedly neglected or faced familial challenges following changes in family circumstances*”.

Eight participants stated that donor privacy is a significant ethical challenge in cross-border fertility travel. They explained that several countries have moved away from anonymous to non-anonymous donation; future children can have access to the donor information. They stated that this may be particularly concerning for Iranian couples who stress confidentiality, especially regarding lineage (nasab, i.e., referring to the legal and religious recognition of parent–child relationships), and inheritance. One of them stated that: “*commercial genetic banks might also compromise privacy even in those countries where anonymous donation systems are in place*”.

Eleven participants described that differences in religious jurisprudence between source and destination countries created dilemmas for couples navigating cross-border reproductive arrangements. For instance, one participant stated that “*Iran’s rules regarding preserving lineage could go against those of the host country*,* thus putting couples into a dilemma while navigating the differences in legal and ethical frameworks*”. Fifteen participants described that the tension between the desire for parenthood and religious considerations may generate ongoing anxiety during the treatment process.

Taken together, participants perceived that psychological distress, stigma, identity concerns, and religious ambiguity interact in a reinforcing cycle that increases uncertainty and social risk, ultimately making secrecy a central coping strategy in cross-border reproductive donation.

### Theme 4. Justice, commodification, exploitation and demand in fertility tourism

Nineteen participants reported that the high cost of treatment, combined with the necessity to travel abroad, limits access to fertility services mainly to couples with greater financial resources. Six participants noted that reproduction and parenthood are increasingly influenced by economic factors and market dynamics. One participant stated: “*the economic disparities between countries have widened*,* with couples from better-off countries visiting Iran and India for treatment*,* as it is cheaper*”. An embryologist explained: “*we frequently see patients from Arab countries coming to Iran for treatment… It’s good for foreign currency to come into the country. One cannot ignore the advantages of fertility tourism. But consider that when they consume these services*,* our resources are limited. They are not infinite.*” Nine participants stated that the presence of foreign patients may reduce access to services for local populations due to limited resources.

Six participants described the involvement of economically disadvantaged women as gamete donors or surrogates as a concerning aspect of reproductive donation. Eleven participants expressed concerns about potential exploitation in these situations. One participant mentioned coercion by husbands and instrumentalization of women’s bodies: “*some women themselves do not want to donate eggs… We have cases here where a husband repeatedly brings his wife*,* holding her hand*,* insisting that she should donate eggs so he can get money.*”

Two participants expressed that the complex networks of consulting agencies and gamete banks mediate between intended parents and treatment centers. They explained that these agencies do much more than medical coordination, and while they might appear helpful, they often remove the control from couples and may cause many kinds of problems for them.

Nine participants suggested that fertility tourism contributes to the development of cross-border markets for gametes, embryos, and surrogacy. Five participants suggested that cross-border reproductive practices may contribute to the commercialization of reproduction. Another participant from the Ministry of Health mentioned: “*the developing market of celebrity gametes*”.

In contrast to the ethical concerns raised, twenty participants expressed a generally positive view toward fertility tourism and acknowledged its growing demand. In fact, “positive perceptions and growing demand for fertility tourism” emerged as a main subtheme. One participant stated: “*fertility tourism is perceived as a practical solution for many couples who otherwise would not have access to such services.*” Participants expressed both concerns regarding ethical and structural issues and a generally positive view toward fertility tourism, alongside increasing demand.

**Fig. 1:**
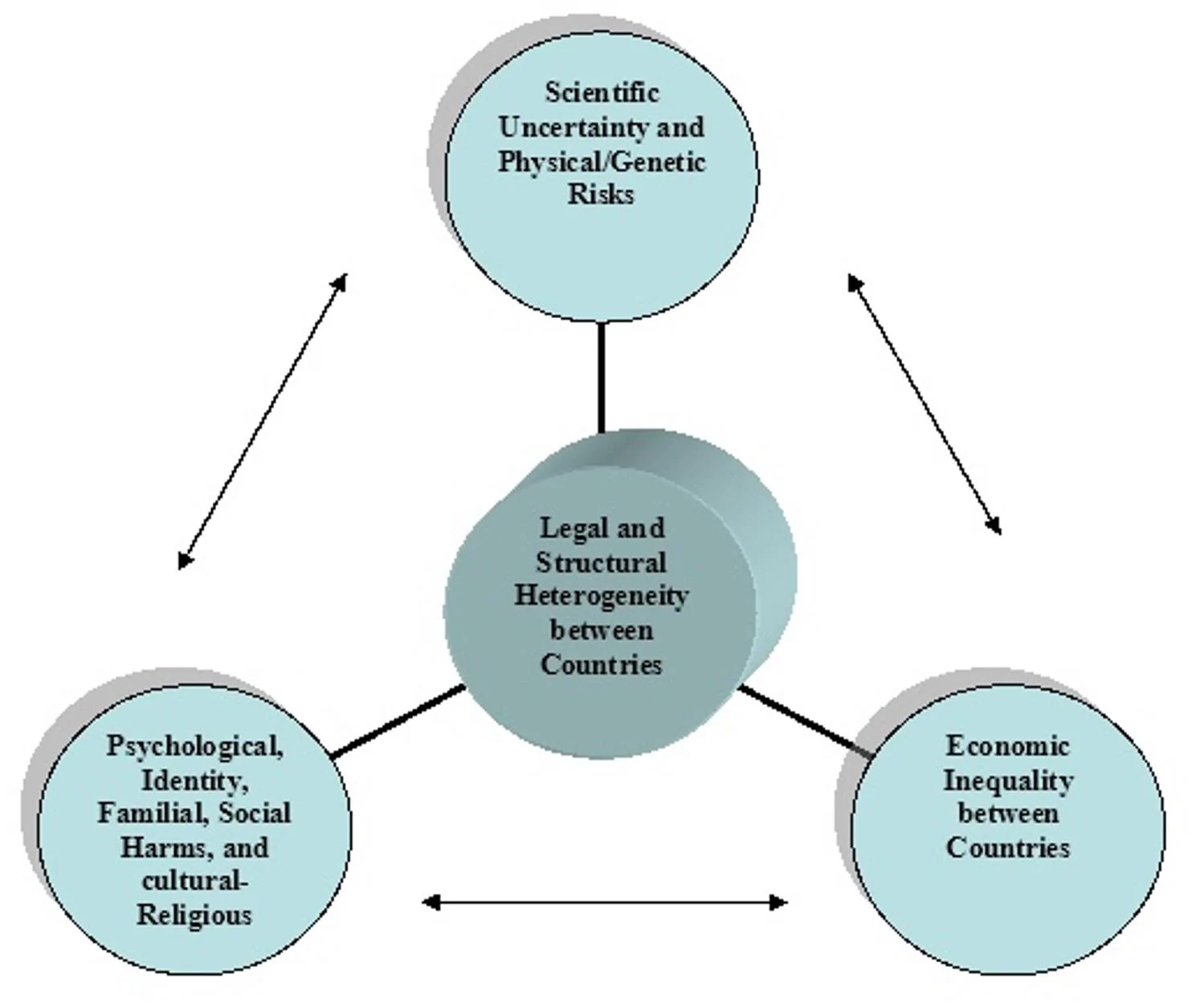
Conceptual model for the challenges of donor-assisted fertility tourism to/from Iran

Ultimately, through repeated and continuous immersion in the data, the researchers found that couples engaging in this form of cross-border reproductive care experience a constant oscillation among interconnected challenges. These challenges may transform into one another or mutually reinforce each other, creating a cyclical and interactive dynamic. Notably, “legal and structural heterogeneity” emerged as the central theme, as all other themes were shaped by, or operated under, its broader influence. In fact, Legal and structural heterogeneity operates as a driver that amplifies ethical risk across legal, medical, social, and economic domains. Therefore, addressing the remaining challenges requires careful consideration of this overarching theme. Each theme can directly or indirectly affect the others, further complicating the landscape of donor-assisted fertility tourism. To enhance conceptual clarity, a conceptual model has been developed (Fig. 1).

## Discussion

This study showed that fertility tourism in donor-assisted reproduction to/from Iran is far from being just a medical or therapeutic process; rather, it involves legal, ethical, cultural, religious, psychological, and economic dimensions. It should be mentioned that a three-level normative framework (state law, Islamic jurisprudence (fiqh), and institutional practice) was used as an analytical lens. Importantly, the study reflects clinicians’ perceptions and interpretations of these overlapping frameworks rather than direct legal analysis. This distinction is essential, as these normative sources may operate with different degrees of authority and may not always be perceived as fully distinct or hierarchically ordered by practitioners, which can contribute to ambiguity in clinical decision-making and ethical reasoning. These findings suggest perceived tensions between autonomy and structural constraints as described by participants.

To further clarify the ethical dimensions of these findings, it is important to distinguish between legal and ethical challenges. While legal challenges refer to formal rules and regulatory frameworks governing reproductive practices, ethical challenges relate to broader principles such as autonomy, justice, beneficence, and non-maleficence. In cross-border reproductive contexts, practices may be legally permissible in one jurisdiction but still raise significant ethical concerns, highlighting the need for careful ethical evaluation beyond legal compliance.

Despite these matters, most of the participants held a positive overall view of fertility tourism related to donors while acknowledging the many challenges involved. No participant recommended abolishing these services, but rather regarded forming regulatory frameworks as a rational and necessary step. This finding is consistent with previous international literature, which has reported that individuals and couples often travel abroad to overcome regulatory barriers or to access more affordable and diverse reproductive options [20, 21]. In this regard, fertility tourism has been increasingly recognized as part of a broader global reproductive market shaped by inequalities in access to assisted reproductive technologies [22]. The findings of the present study align with this global pattern, suggesting that despite ethical and structural concerns, demand for donor-assisted fertility services persists and may continue to grow.

Findings showed that donor-based fertility tourism involving Iran was a complex and multi-faceted phenomenon, consisting of four main themes and seventeen subthemes, each with distinct challenges. Participants suggested that cross-border differences in regulation may contribute to perceived geopolitical challenges. It should be noted that many of the legal and geopolitical implications reported in this study reflect clinicians’ perceptions and anecdotal experiences rather than systematically verified legal outcomes. From an ethical perspective, these legal inconsistencies raise concerns related to justice and equity, as unequal regulatory environments may lead to unequal access to services and place certain groups at a disadvantage. In addition, differences in laws regarding gamete donation, embryo donation, and surrogacy between countries of origin and destination cause confusion, birth registration and child citizenship problems, and procedural problems for those returning with the child. Anastasiadou and colleagues (2025) also found that foreign birth certificates are not automatically recognized in Greece and thus require a new certificate to be issued by the embassy; couples are often obliged to legally adopt their child born through surrogacy [14]. In Slovenia, women who intend to relinquish a child to a third party-a surrogate mother-are not allowed access to ART, and IVF treatments must be done using the gametes of at least one of the parents; it is not allowed to use donated gametes from both parents [23].

Consistent with this complexity, Yang (2020) has termed this type of travel as “circumvention tourism”, wherein patients travel to other countries to seek medical services that are legally permitted in the country of destination but are forbidden in their own country [5]. For example, one of the participants in our research perceived that restrictions related to treatment of single individuals is banned in Iran, and thus forces such people to go to countries such as Ukraine or Russia.

In addition, the lack of standardized administrative and managerial protocols in fertility centers leads to delays, errors in data processing, and high administrative workload. Documentation exchange between the countries of origin and destination was also highlighted as a priority concern. The result is consistent with the previous studies identifying that discrepancies in the systems of data registration and reporting place undue psychological and legal burden on the families and point to the development of uniform national and international legislation [24, 25].

Other challenges that were mentioned, though not the most dominant, were scientific and medical. They referred to the lack of a unified scientific protocol in Iran, different ideas about the quality of the gametes, and the number of donation cycles permissible, which could lead to clinical ambiguity. Participants perceived that such ambiguity might increase concerns regarding donor safety and possible future genetic or demographic implications. These concerns reflected participants’ anticipatory views and should not be interpreted as evidence of documented population-level outcomes. Chen and colleagues (2020) have also pointed out that sex selection through donation, now a motivation for approaching ART, may contribute to gender imbalance, reflected in some clinicians’ concerns in the present study, particularly in countries like China with longstanding one-child policies [9]. These challenges may influence clinical decision-making by creating ambiguity in treatment choices, variability in practice across centers, and uncertainty in patient counseling. Beyond technical considerations, the lack of standardized scientific protocols also represents an ethical concern, as it may compromise the principles of beneficence and non-maleficence by increasing the risk of harm to donors and reducing the quality and safety of care.

Another major theme that emerged in the analysis involved psychological, identity-related, familial, and social consequences of fertility tourism. These include the confusion of identity among donor-conceived children, anxiety among parents, and a significant amount of family tension. This also aligns with psychological research indicating that donor-conceived offspring stand to encounter mental instability [26, 27]. To illustrate this, Marinelli and colleagues (2025) identified in Italy that surrogacy may be perceived as deteriorating the dignity of surrogate mothers and further placing the donor-conceived offspring in more vulnerable situations compared to children born to mothers who deliberately raise them. Correspondingly, through Law No. 169, Italy criminalizes surrogacy even when carried out abroad, provided at least one intended parent is an Italian citizen [26].

A striking finding of the present study was the synergy of three layers of stigma: stigma surrounding infertility, stigma surrounding donation, and stigma attached to the concept of “tourism.” Participants explained that infertility remains a taboo, while using another person’s gametes or uterus is likewise stigmatized; in addition, the term “tourism” evokes assumptions of leisure and pleasure, further intensifying societal judgment. Kraljić (2020) also emphasized that the term “tourism” itself can be stigmatizing, suggesting that a term such as “cross-border reproductive transactions” or “reproductive exile” may be more fitting [17]. HashemiNasab and Garousi (2024) described surrogacy in Iran as embedded within a complex network of social and family expectations, and cultural pressures surrounding childbearing [15]. Likewise, Ghane-Mokhallesouni et al. (2021) reported that surrogate mothers experienced stigma, emotional strain, and social judgment within their communities [16]. In our study, this triple stigma led couples to conceal the method of conception even from close family members, thus creating an ethical and religious dilemma: whereas they wanted to keep the secret, religious teachings emphasize the child’s right to identity, referring to the right to know one’s genetic and familial origins.

As Mutlu (2024) also mentioned, stigma forces egg donors in Turkey to hide, making them even more susceptible to physical harm and exploitative practices. In describing this pattern, she termed it self-harm [28]. Concerns over the donor-conceived children’s future in foreign countries regarding their fate following their social parents’ death further complicate decision-making for parents. Also, the debate on whether anonymity should be maintained for donors or non-anonymous donation should be practiced becomes stronger in cross-border contexts. Some countries are also attempting to move towards open-identity donation, which may contradict Iranian couples’ reasons focusing on maintaining confidentiality for lineage and inheritance. Even in settings where anonymity is still legally protected, commercial genetic databases can compromise privacy [29].

Another critical challenge involved economic inequality and the global commercialization of reproduction. High treatment and travel costs restrict access primarily to affluent groups, while low-income women may be vulnerable to exploitation. Cross-border markets for gametes and surrogacy transfer decision-making power to economic factors rather than ethical or medical principles [30]. Historically, patients from low and middle-income countries traveled to high-income countries for better medical services [31]; however, in reproductive tourism, this pattern is often reversed, particularly for surrogacy [29], with wealthier patients traveling to less affluent countries due to legal restrictions or personal circumstances [27]. This trend mirrors broader economic inequalities between consumers and providers [32]. As one participant poignantly put it, “creating a child requires nothing more than internet access and a sufficiently funded credit card,” vividly highlighting the commodification of reproduction. This global market may raise concerns regarding practices that challenge basic bioethical principles prohibiting the sale of body parts [33, 34].

According to Zhukova (2025), high ART costs make infertility primarily an issue of privileged populations, while economically disadvantaged individuals are silenced [35]. Low-income women even go abroad and sell their oocytes-a process referred to by Tober and Kroløkke (2021) as “reproductive colonialism” in the post-colonial era [4]. Similarly, Chen (2025) described how Taiwanese women’s oocytes are sold abroad despite the prohibition by national law [36]. For Barat (2023), this aggressive marketing has resulted in unethical and unsafe medical practices in surrogacy tourism in India [37]. These findings are like the subtheme of economic inequity identified in our study.

While the issue may be far from immediately apparent, the growth of such markets eventually leaves the low-income segments of women and men outside the reach of such medical assistance, which may raise serious ethical concerns in medical ethics [38]. In fact, participants expressed concerns related to the principle of justice in medical ethics, as access to treatment becomes dependent on financial capacity rather than medical need. The involvement of economically disadvantaged women as donors or surrogates, particularly in situations suggestive of coercion, challenges the principle of respect for autonomy and raises serious concerns regarding exploitation and informed consent [3, 39]. Furthermore, the increasing commercialization of reproductive services may conflict with ethical principles that prohibit the commodification of the human body [11].

The findings also highlight the gendered dimensions of cross-border reproductive donation and fertility tourism. Participants perceived that women disproportionately bear the physical, emotional, and social burdens associated with reproductive donation, particularly in relation to egg donation and surrogacy. Concerns regarding repeated ovarian stimulation, physical complications, and the involvement of economically disadvantaged women reflected broader ethical concerns about reproductive labour and the commodification of women’s bodies. In addition, some participants described situations in which women’s reproductive decisions appeared to be influenced by economic dependency, marital expectations, or family pressure. These findings raise important questions regarding the extent to which consent can be considered fully autonomous under conditions of financial vulnerability and gendered power imbalance.

The findings further suggest that reproductive tourism may be shaped by broader gender norms surrounding infertility, motherhood, and family stability. Participants described cases in which women sought reproductive donation to preserve marital relationships or avoid social stigma associated with infertility. This aligns with previous literature emphasizing that reproductive labour and cross-border reproductive arrangements are often embedded within unequal social, economic, and gender structures [40, 41]. Therefore, ethical evaluation of fertility tourism should not be limited to legal and clinical considerations alone, but should also consider gendered inequalities and the social conditions influencing reproductive decision-making.

Taken together, these findings suggest that donor-assisted fertility tourism encompasses a far broader scope than is generally assumed. Balancing intended parents’ needs and financial capabilities with ethical values and the rights of donor-conceived children in countries of origin and destination, along with the demographic interests of governments, is a profoundly challenging task. The medical, legal, bioethical, psychological, sociological, and related professions must pay more attention to this emerging area and work together to establish consistent national and international regulations.

These findings can also be interpreted considering international bioethical frameworks. In this relation to the UNESCO Universal Declaration on Bioethics and Human Rights underscores respect for human dignity, autonomy, informed consent, the right to identity, and the protection of vulnerable groups [42]. The findings of this study, especially concerns regarding donor anonymity, identity disclosure, and the psychological implications for donor-conceived children, align with the Declaration’s emphasis on the child’s right to identity, while simultaneously highlighting practical challenges in implementing this principle in cross-border reproductive arrangements.

In relation to the Oviedo Convention, which emphasizes the primacy of the human being, and the prohibition of financial gain from the human body [43], several of the ethical concerns identified in this study—particularly those related to the commodification of gametes and reproductive services—appear to be partially aligned with these principles.

Furthermore, ongoing discussions within the Hague Conference on Private International Law regarding international surrogacy and parentage reflect concerns about legal parentage, citizenship, and the absence of harmonized international regulations [44]. The legal and structural heterogeneity identified in this study mirrors these global challenges, demonstrating how inconsistencies between jurisdictions can lead to uncertainty in citizenship, parentage recognition, and the legal status of donor-conceived children.

From a medical ethics perspective, the findings of this study highlight several key ethical principles that are challenged in the context of reproductive donation tourism. The legal and structural heterogeneity identified by participants raises concerns related to justice and equity, as unequal access to services and inconsistent regulations may disadvantage certain groups while privileging others.

The reported medical uncertainties and potential physical harms to donors point to issues of non-maleficence and the obligation to minimize harm in clinical practice. In addition, the lack of standardized protocols in Iran may compromise beneficence by affecting the quality and safety of care.

The psychosocial and identity-related challenges described by participants—particularly those related to stigma, secrecy, and the rights of donor-conceived children—highlight tensions between confidentiality and the child’s right to identity, as well as broader concerns related to human dignity and psychological well-being. These findings raise important ethical concerns regarding the balance between confidentiality and the child’s right to identity, reflecting a tension between respect for parental privacy and the ethical obligation to support transparency and the long-term psychological well-being of donor-conceived individuals.

Finally, the commercialization of reproductive services and the potential exploitation of economically vulnerable women raise serious ethical concerns regarding justice, autonomy, and the commodification of the human body. These findings underscore the need for ethically grounded regulatory frameworks that go beyond legal permissibility and address the broader moral implications of cross-border reproductive practices. These matters highlight key ethical challenges related to justice, autonomy, and human dignity. Unequal access to services raises concerns about fairness, while the involvement of economically vulnerable women in donation or surrogacy arrangements may undermine genuine informed consent and increase the risk of exploitation.

Therefore, taken together, these findings have several practical and policy implications for fertility tourism and reproductive donation governance. Participants’ concerns regarding donor safety and clinical uncertainty highlight the need for standardized national and cross-border protocols for donor screening, limits on repeated donation cycles, long-term medical follow-up, and harmonized clinical guidelines across ART centers. In addition, the findings suggest the importance of independent psychological, ethical, and legal counselling for donors and recipients before treatment, particularly in cross-border arrangements where differences in legal, religious, and cultural frameworks may increase uncertainty. Participants’ accounts of potential coercion and economic vulnerability further indicate the need for stronger safeguards to protect donors and surrogates, including transparent consent procedures and independent monitoring mechanisms. The findings also support the development of clearer policies regarding donor anonymity, donor-conceived children’s rights to information about biological origins, and international mechanisms for cross-border data registration and documentation. Finally, greater transparency and regulation of intermediary agencies and gamete banks may help reduce exploitation, misinformation, and unequal power dynamics within the fertility market.

This study has several limitations. First, donors and recipients in cross-border fertility processes were not directly involved out of respect for their privacy and emotional burden. The results therefore present the views of professionals and policymakers in the field of infertility care. Such research in the future that involves individuals with lived experience would offer a deeper understanding of their real needs, difficulties, and expectations. In addition, this study does not provide a legal or jurisprudential analysis of Iranian reproductive frameworks; rather, it reflects clinicians’ interpretations of how state law, fiqh, and institutional regulations interact in practice.

## Conclusion

The findings of this study suggest that donor-assisted fertility tourism is a very challenging experience with a multidimensional and deeply interrelated character, whereby legal, ethical, cultural, religious, psychological, social, economic, genetic, and demographic factors interact in a cyclic and self-reinforcing way. The results also highlight that reproductive donation tourism is not merely a legal and clinical issue but one that raises significant ethical concerns, requiring careful consideration of fundamental principles of medical ethics in both national and international policy-making. Participants’ perceptions suggest that these consequences may extend from individuals and families to broader societal domains such as health systems, legal structures, and, in some cases, national-level considerations. The results suggest the importance of developing harmonized legal frameworks, common scientific standards, ethical guidelines, and easily available psychological and religious counseling. Overcoming the steadily growing challenges facing fertility tourism urges close collaboration between medical professionals, legal scholars, bioethicists, psychologists, and health policy experts.

## Supplementary Information

Supplementary Material 1.

## Data Availability

The datasets are available from the corresponding author on reasonable request.
