# Supplementary material for: Ethical challenges in reproductive donation tourism: a qualitative study of clinicians’ perspectives in Iran
Source: BMC Med Ethics. 2026 Jul 1;27:210. doi: 10.1186/s12910-026-01547-8 (PMC13625292; doi:10.1186/s12910-026-01547-8)
Supplement: Supplementary file 1 [file 12910_2026_1547_MOESM1_ESM.docx]

**Interview Guide**

## Purpose

This guide is intended for the interviewer only. It provides instructions, suggested prompts, and probes to ensure consistent and comprehensive data collection during semi-structured interviews.

The goal is to explore clinicians’ perceptions of ethical challenges in donor-assisted reproductive tourism, including legal, medical, psychological, social, and cultural dimensions.

## Interview instructions

1. **Before the interview:**

- Explain the purpose of the study to participants.
- Reassure participants that their responses are confidential and voluntary.
- Obtain written informed consent and permission to audio-record.

2. **During the interview:**

- Begin with broad, open-ended questions.
- Use probes to explore emerging ideas or clarify ambiguous statements.
- Maintain a neutral, non-judgmental tone.
- Avoid leading questions.
- Allow pauses and silence; encourage participants to elaborate.
- Document non-verbal cues.
- Be flexible with question order.
- **Suggested questions**:

1. Since you began working in the field of infertility, what have you seen or heard regarding third-party reproductive tourism?
2. When you think about reproductive tourism involving third-party donation, what are the concerns that first come into your mind?
3. Do these concerns relate to legal, ethical, or social aspects?
4. What makes you to feel worried or uncertain about this treatment?
5. What challenges and barriers have you faced relating to reproductive tourism involving third-party donation?

- **Probes**:

Can you give specific examples?

Please more explains?

- **Closing question**:

Do you have any additional comments or reflections regarding reproductive tourism or third-party donation?”

3. **After the interview:**
   - Thank the participant.
   - Check if they have any additional comments.
   - Ensure that all notes and recordings are securely stored.
